# Protective Effects of Nongxiang‐Type *Tieguanyin* on the Liver of D‐Galactose‐Induced Aging C57BL/6 Mice via the NF‐κB Signaling Pathway

**DOI:** 10.1002/fsn3.72396

**Published:** 2026-09-29

**Authors:** Weihong Chen, Mingmei Li, Lingxiao Ye, Dongqin Huang, Jianjia Xiao, Yuchao Sang, Zizhen Wang, Jiehong Yang, Chengyong He, Jiabin Chen, Zhenghong Zuo, Xintan Chen

**Affiliations:** ^1^ Department of Central Laboratory Anxi County Hospital Quanzhou Fujian China; ^2^ State Key Laboratory of Cellular Stress Biology, School of Life Sciences, Faculty of Medicine and Life Sciences Xiamen University Xiamen Fujian China; ^3^ Anxi County Center for Disease Control and Prevention Quanzhou Fujian China

**Keywords:** attenuation of D‐gal‐induced senescence, hepatoprotection, Nongxiang‐type *Tieguanyin*, oxidative stress

## Abstract

This research aimed to investigate the hepatoprotective and anti‐senescence effects of Nongxiang‐type Tieguanyin (Tgy‐N) on D‐galactose (D‐gal)‐induced aging C57BL/6 mice and to elucidate the underlying mechanisms. The chemical composition of Tgy‐N was characterized by colorimetric assays and HPLC. An aging mouse model was established by daily intraperitoneal injection of D‐gal (200 mg/kg) for 8 weeks, with simultaneous intragastric administration of Tgy‐N at 110 or 550 mg/kg/day. Behavioral tests, serum biochemical analyses, hepatic oxidative stress markers, histopathological examination, transcriptomic profiling, and western blotting were performed. The anti‐senescence effect was further validated in Nutlin‐3a‐induced senescent AML12 and HepG2 cells. Tgy‐N contained abundant tea polyphenols (286.91 mg/g) and catechins (137.89 mg/g). HPLC analysis with authentic external standards revealed that the absolute contents of EGCG, catechin, catechin gallate, gallic acid, and epigallocatechin in Tgy‐N extract were 7.96, 5.79, 4.06, 1.67, and 4.91 mg/g, respectively. In vivo, Tgy‐N significantly improved exercise endurance and energy metabolism, reduced the hepatosomatic index, attenuated hepatic oxidative stress (elevated SOD and GSH, reduced MDA), and decreased serum ALT and AST levels. Histologically, Tgy‐N ameliorated hepatic lobule disorganization, reduced fibrosis, decreased SA‐β‐gal‐positive areas, downregulated P16/P21/P53, and upregulated Sirt1. Transcriptomic and western blot analyses revealed that Tgy‐N suppressed the NF‐κB signaling pathway by reducing phosphorylation of IKKα/β and p65. Cellular experiments confirmed that Tgy‐N reduced p16/p21 expression and SA‐β‐gal‐positive cell numbers in Nutlin‐3a‐induced senescent hepatocytes. Tgy‐N protects against D‐gal‐induced hepatic senescence through oxidative stress mitigation and NF‐κB pathway inhibition, supporting its potential as a natural functional food for liver health.

## Graphical abstract

Mechanistic diagram of the hepatoprotective effect of Tgy‐N on D‐gal‐induced senescent C57BL/6 mice (“→” indicates activation, and “**⊣**” indicates inhibition).

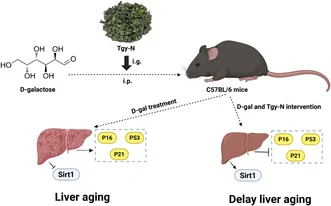

## 1 Introduction

*Tieguanyin*, a representative oolong tea in China, is produced in Anxi, Fujian Province. Based on processing techniques, it is classified into Nongxiang type (Tgy‐N), Qingxiang type, and other types (Zheng et al. 2025). Among them, the Tgy‐N undergoes further processing through roasting technology, resulting in flavor and bioactive compound profiles that differ markedly from other Tieguanyin types (Zeng et al. 2025). During the roasting process, not only are the unique flavors of Tgy‐N (such as caramel and jujube aromas) formed, but also the structure and content of its active ingredients are altered, thereby providing the material basis for its unique physiological functions, including antioxidant, anti‐inflammatory, metabolic regulation, and cardiovascular protection effects (Su et al. 2025). The extract of Tgy‐N has been shown to reduce the levels of differential fecal metabolites in Alzheimer's disease (AD) model mice and modify the gut microbiota structure by targeting a variety of bacteria; meanwhile, it contributes to altering the fecal metabolite profile of AD mice, with its regulatory effects on metabolites and gut microbiota potentially providing a basis for exploring AD‐related diagnostic biomarkers (Tu et al. 2022).

Aging is a natural process in which organisms gradually transition from maturity to decline. Its essence is a comprehensive manifestation of the gradual functional degradation and structural damage of the body's cells, tissues, and organs at the molecular, cellular, and organ levels (Cai et al. 2025). With the intensification of the global population aging trend, the incidence of aging‐related diseases has been increasing year by year (Wang et al. 2025). This not only seriously affects the quality of life of the elderly but also imposes a heavy burden on the social medical system (Tang et al. 2025). Therefore, in‐depth analysis of the physiological mechanism of aging and the exploration of effective methods to delay aging and intervene in aging‐related damage have become research hotspots in the fields of modern medicine and life sciences (Tipparaju et al. 2025).

Aging exerts a significant impact on various systems and organs of the body, manifested as decreased immune function, weakened digestive function, reduced elasticity of the cardiovascular system, and impaired cognitive function of the nervous system (Golubev 2025). Among these organs, the liver, as the largest metabolic and detoxification organ of the body, is closely related to the health of the body and has become one of the organs most vulnerable to damage during the aging process (Williams and Ding 2025). A series of structural and functional changes occur in the liver during aging (Zhong et al. 2025). These changes not only affect the physiological functions of the liver itself but also further accelerate the aging process of the body through pathways such as metabolic disorders and decreased detoxification capacity (Ajoolabady et al. 2025). Therefore, studying the mechanism of liver aging and exploring effective protective methods are of great practical significance for delaying body aging and preventing aging‐related liver diseases (Husain et al. 2025; Ganguly et al. 2025).

In this research, we first characterized the chemical composition of Tgy‐N using colorimetric assays and HPLC. We then established a D‐gal‐induced aging mouse model and administered Tgy‐N at 110 or 550 mg/kg/day by gavage for 8 weeks. Focusing on the liver, we performed behavioral assessments, serum biochemical analyses, oxidative stress marker detection, histopathological examination, transcriptomic profiling, and western blotting to evaluate the protective effects of Tgy‐N and elucidate its mechanistic basis. In addition, Nutlin‐3a‐induced senescent AML12 and HepG2 cells were employed to validate the anti‐senescence effect of Tgy‐N in vitro. This study aims to provide a theoretical and experimental foundation for the application of Tgy‐N in mitigating D‐gal‐induced senescence and to promote its development from a traditional beverage into a natural functional food or anti‐aging agent.

## 2 Materials and Methods

### 2.1 Materials

#### 2.1.1 Experimental Animals

Male C57BL/6 mice were chosen as the experimental animals. All the selected mice were accommodated in the Experimental Animal Center of Xiamen University, where the ambient temperature was regulated to 22°C ± 2°C, the relative humidity was kept at 50% ± 10%, and a 12‐h light/12‐h dark circadian cycle was maintained. The mice were provided with unrestricted access to food and water to sustain their normal physiological conditions. The present research protocol was reviewed and approved by the Animal Ethics Committee of Xiamen University (approval number: XMULAC20170361).

#### 2.1.2 Experimental Drugs and Reagents

Tgy‐N was supplied from Bama Tea Co. Ltd.; D‐Galactose (CAS 59‐23‐4) was purchased from MCE. Kits (including serum aspartate aminotransferase [AST], alanine aminotransferase [ALT], superoxide dismutase [SOD] [A001‐1‐1], malondialdehyde [MDA] [A003‐1‐2], glutathione [GSH] [A006‐2‐1], etc.) were purchased from Nanjing Jiancheng Bioengineering Institute. Mouse anti P16 (ZMS1072‐25, 1:1000), Mouse anti P53 (ab90363, 1:1000), and Rabbit anti Sirt1 (ab189494, 1:1000) were purchased from Abcam; Mouse anti α‐sma (ABM0052, 1:1000) was purchased from Abbkine; HRP‐conjugated goat anti‐mouse IgG (SA00001‐1; 1:5000), and goat anti‐rabbit IgG (RGAR001; 1:5000) were purchased from Proteintech; Rabbit anti NF‐κB (A2445, 1:1000), Rabbit anti Phospho‐NF‐κB (AP0490, 1:1000), Masson staining reagents (Cat#G1346) were purchased from Solarbio. β‐gal staining reagents (RG0036‐3) were purchased from Beyotime.

#### 2.1.3 Experimental Instruments

Rodent Treadmill NG (Ugo Basile, 47300), Grip strength meter (Ugo Basile, 47200), Promethion Line system (CAB‐16‐1‐EU, Sable Systems), Rotary evaporator (Rotavapor R‐300, BUCHI), Perfection V850 Pro scanner (Epson).

### 2.2 Methods

#### 2.2.1 Preparation of Tgy‐N Extract

Dried Tgy‐N was fully pulverized using a crusher. A suitable amount of the pulverized Tgy‐N was weighed out and added to boiling water for the extraction process. The initial extraction was carried out for 30 min under constant stirring to ensure thorough dissolution of bioactive components into the aqueous phase. Following the first extraction, vacuum filtration was conducted to collect the resulting filtrate. The residual tea dregs were then mixed with fresh boiling water for a second extraction, which lasted 20 min. After filtering the second extract, the two batches of filtrate were combined. The merged filtrate was concentrated using a rotary evaporator; this device facilitated water evaporation, reducing the filtrate volume to roughly one‐tenth of its original size. The concentrated solution was transferred to a −20°C refrigerator for a 2‐h incubation period, after which freeze‐drying was performed to obtain Tgy‐N extract dry powder. It was hermetically sealed and stored in a desiccator for subsequent experimental use.

#### 2.2.2 Determination of Chemical Components of Tgy‐N

Tea polyphenols were determined using the Folin method (Matsushita et al. 2024): briefly, 0.2 g of ground tea sample was extracted twice with pre‐warmed 70% methanol, and the mixture was centrifuged after each extraction; the resulting supernatants were combined, filtered, and diluted. 1 mL aliquot of the diluted test solution was mixed with 5 mL of 10% Folin reagent and reacted for 3–8 min, followed by the addition of 4 mL of 7.5% Na_2_CO_3_; the mixture was allowed to stand at room temperature for 60 min, and its absorbance was measured at 765 nm. Flavonoids were quantified via the AlCl_3_ colorimetric method (Pasca et al. 2025): 0.9 g of ground tea was extracted with 100 mL of boiling water for 45 min and filtered, then 1 mL of the extract was mixed with 9 mL of 1% AlCl_3_ solution and reacted at 25°C for 10 min, with 1% AlCl_3_ solution used as the blank and absorbance measured at 420 nm. Tea polysaccharides were analyzed by the anthrone‐sulfuric acid method (Ren et al. 2023): 0.001 g of the sample was mixed with 10 mL of distilled water, 1 mL of this mixture was combined with 4 mL of 2 g/L anthrone‐sulfuric acid solution, heated at 100°C for 10 min, cooled, and its absorbance was measured at 620 nm. Caffeine and catechins were quantified spectrophotometrically (Nanjegowda et al. 2016): for caffeine, 3 g of ground tea was extracted with boiling water for 45 min and filtered; a 10 mL aliquot of the extract was mixed with 4 mL of 0.01 mol/L HCl and 1 mL of basic lead acetate, diluted, and filtered; 25 mL of the subsequent filtrate was treated with 0.1 mL of 4.5 mol/L H_2_SO_4_, further diluted, filtered, and its absorbance was measured at 274 nm; for catechins, 0.2 g of ground tea was extracted with 70% methanol, centrifuged, and the supernatant was filtered, diluted, and its absorbance was measured at a specific wavelength. Theaflavins, thearubigins, and theabrownins were quantified via spectrophotometry (Xu et al. 2023): 3 g of tea sample was extracted with boiling water and filtered; the extract was partitioned with ethyl acetate and *n*‐butanol to prepare solutions; with 95% ethanol as the blank, the absorbance of these solutions was measured at 380 nm for content calculation. Free amino acids were determined using the ninhydrin method (Ofitserova and Nerkar 2016): 3 g of ground tea was extracted with boiling water for 45 min and filtered; 1 mL aliquot of the extract was mixed with 0.5 mL of pH 8.0 phosphate buffer and 0.5 mL of 2% ninhydrin solution, heated in a boiling water bath for 15 min, cooled, and its absorbance was measured at 570 nm.

#### 2.2.3 HPLC Analysis of Catechin Components in Tgy‐N

The catechin and gallic acid (GA) profiles of Tgy‐N extract were analyzed by high‐performance liquid chromatography (HPLC) according to GB/T 8313‐2018. Briefly, the extract powder was extracted with 70% methanol, and the supernatant was filtered and injected onto a C18 column (4.6 × 150 mm, 5 μm) at 35°C, with gradient elution using 0.1% formic acid in water and acetonitrile as the mobile phase at 1.0 mL/min. The gradient program was as follows: 0–5 min, 5% acetonitrile; 5–15 min, 5%–20% acetonitrile; 15–25 min, 20%–30% acetonitrile; 25–30 min, 30%–5% acetonitrile. Detection was performed at 280 nm, and individual components were identified by comparison of retention times with authentic standards, with quantification based on peak area percentage normalization. The shorter column (150 mm) and optimized gradient program were employed to improve analytical throughput, which resulted in earlier elution of polar compounds such as GA and EGCG. All analyses were performed in triplicate. The method validation parameters, including linearity, LOD, LOQ, and *R* ^2^ for each analyte, are summarized in Table S1. For quantitative analysis using authentic external standards, an additional HPLC method was performed on an Agilent Zorbax SB‐C18 column (4.6 × 250 mm, 5 μm) at 30°C, with a flow rate of 1.0 mL/min and detection at 280 nm. The mobile phases consisted of 0.1% formic acid in water (A) and acetonitrile (B). The gradient program was: 0–10 min, 10% B; 10–20 min, 10%–20% B; 20–30 min, 20%–30% B; 30–35 min, 30%–10% B; followed by re‐equilibration. Quantification of EGCG, C, CG, GA, and EGC was performed using authentic external standards with calibration curves constructed from five concentration levels (Table S1).

#### 2.2.4 Establishment of Mouse Aging Model and Grouping Treatment

The aging model in mice was established via intraperitoneal administration of D‐gal. C57BL/6 mice were randomly assigned to four experimental groups, with 10 mice per group: the control group, the model group, the Tgy‐NL (low‐dose) group, and the Tgy‐NH (high‐dose) group. Treatments for each group were as follows: the control group received intraperitoneal injections of 0.9% sodium chloride (NaCl) and intragastric water; the model group was given intraperitoneal injections of D‐gal at a dose of 200 mg/kg/day, with intragastric water; the Tgy‐NL group received intraperitoneal D‐gal at a dose of 200 mg/kg/day, with intragastric Tgy‐N at 110 mg/kg/day; the Tgy‐NH group received intraperitoneal D‐gal at a dose of 200 mg/kg/day, with intragastric Tgy‐N at 550 mg/kg/day. All treatments were administered once daily for a continuous 2‐month period. In the present research, the selected Tgy‐N doses (110 mg/kg and 550 mg/kg for mice) are equivalent to approximately 5 g and 25 g of Tgy‐N per day for a 60 kg human, respectively. The dose selection was based on the following considerations. The low dose (110 mg/kg) was calculated from the typical daily tea intake of Chinese adults (approximately 5 g of dry tea leaves per day, equivalent to ~83 mg/kg for a 60 kg human). Using the allometric scaling method based on body surface area, the Km factor ratio between mouse and human is 12.3, which is a widely accepted conversion factor in pharmacological studies. The calculated mouse equivalent dose is approximately 110 mg/kg. Moreover, this dose falls within the typical dose range of tea polyphenols (100–400 mg/kg) used in mouse anti‐aging studies. The high dose (550 mg/kg) was set at five times the low dose to evaluate potential dose‐dependent effects (Nair and Jacob 2016).

#### 2.2.5 Behavioral Experiments

##### 2.2.5.1 Treadmill Test

Following 2 months of treatment, treadmill testing was conducted on the mice. Prior to the formal test, the mice underwent training on the treadmill at a speed of 10 m/min for 5 min per session for 2 consecutive days. A 2‐h fasting period was implemented before the official experiment. For the test itself, the treadmill was adjusted to a 15° incline, and its speed was raised gradually from 10 to 18 m/min. The fatigue threshold was defined as the accumulation of ≥ 5 electric shocks (0.1 mA) within 2 consecutive minutes, and the total running distance of each mouse was recorded.

##### 2.2.5.2 Grip Strength Test

Each mouse was secured by its tail, enabling its limbs to grasp a T‐shaped bar linked to a grip strength meter. The mouse was then pulled away at a steady speed until its limbs completely released the bar. This procedure was repeated 5 times, with a 5‐min rest interval between each trial. The average value of the maximum grip force recorded across the trials was used as the final result to assess the mice's muscle strength.

##### 2.2.5.3 Rotarod Fatigue Test

Mice were placed individually on a rotating rod set to an initial speed of 20 rpm. The time elapsed until each mouse fell off the rod was recorded. Each mouse was tested 3 times, and the average time from these trials was taken as the final result to evaluate coordinated motor function and anti‐fatigue capability.

##### 2.2.5.4 Metabolic Cage Experiment

A Promethion Line system (model CAB‐16‐1‐EU, supplied by Sable Systems) was used to measure the energy expenditure (EE) of the mice. Each of the 16 metabolic cages was equipped with standard bedding, unrestricted access to food and water, and load cells for monitoring purposes. Before the experiment, the mice were acclimated in gas‐calibrated chambers for 1 week. They were then randomly assigned to their respective groups, and their body weight and fat ratio were measured both before and after the acclimation period. Daily food intake was tracked throughout the experiment, and mice that failed to acclimate were excluded from the analysis. Data were collected at 5‐min intervals using MetaScreen software (version 2.3.15.12) and analyzed with Macro Interpreter software (version 2.32). XYZ beam arrays (with 0.25 cm spacing) were used to monitor the mice's activity levels. Respiratory gases were measured at a flow rate of 2000 mL/min using a GA‐3 analyzer, and the values of oxygen consumption (VO_2_) and carbon dioxide production (VCO_2_) were reported in mL/min (with water vapor effects compensated for). Energy expenditure was calculated using the Weir equation: [kcal/h = 60 × (0.003941 × VO_2_ + 0.001106 × VCO_2_)]. Analysis of covariance (ANCOVA) was applied to compare average EE differences between groups, with body weight included as a covariate.

#### 2.2.6 Collection and Detection of Organ Samples

##### 2.2.6.1 Organ Sample Collection

At the conclusion of the experiment, the mice were euthanized, and organ samples (including the liver, heart, lung, and kidney) were collected promptly. Each organ was rinsed with physiological saline to remove surface blood, blotted dry with absorbent material, and weighed. The ratio of organ weight to body weight was then calculated for each mouse.

##### 2.2.6.2 Detection of Serum Biochemical Indicators

Blood samples were collected from the orbital sinus of each mouse into centrifuge tubes. The tubes were allowed to stand at room temperature for 30 min, after which they were centrifuged at 3000 rpm for 15 min to separate the serum. The levels of AST and ALT in the serum were detected using the corresponding commercial kits, following the manufacturers' recommended protocols.

##### 2.2.6.3 Detection of Hepatic Oxidative Stress Indicators

A suitable amount of mouse liver tissue was excised and mixed with physiological saline to prepare a 10% liver tissue homogenate. The levels of SOD, MDA, and GSH in the liver homogenate were measured using the respective assay kits, in accordance with the instructions provided by the kit manufacturers.

#### 2.2.7 Liver Transcriptomic Analysis

Mouse liver tissue samples were collected, and total RNA was extracted from these tissues using an RNA extraction kit, following the manufacturer's protocol provided with the kit. The quality and concentration of the isolated RNA were assessed via agarose gel electrophoresis and a UV–Vis spectrophotometer; these steps ensured that the RNA's integrity and purity met the criteria required for transcriptome sequencing.

The extracted RNA was then used to construct sequencing libraries, which involved a series of steps: RNA fragmentation, reverse transcription, cDNA synthesis, end repair, A‐tailing, and adapter ligation. Once the constructed libraries passed quality verification, high‐throughput sequencing was performed on a dedicated sequencing platform to generate transcriptomic data for the mouse liver tissues.

The raw sequencing data underwent quality control and filtering processes to eliminate low‐quality reads and adapter sequences. Subsequent analyses—including sequence alignment, gene expression quantification, differential gene identification, Gene Ontology (GO) enrichment analysis, Kyoto Encyclopedia of Genes and Genomes (KEGG) enrichment analysis, and Gene Set Enrichment Analysis (GSEA) were conducted using relevant bioinformatics software. Differential genes were screened based on the thresholds of *p* < 0.05 and |Log_2_FC| > 1, followed by analysis of the functions of these differential genes and the signaling pathways they are involved in.

#### 2.2.8 Liver Histopathological Examination

##### 2.2.8.1 Tissue Processing and Sectioning

Mouse liver tissues were fixed in 4% paraformaldehyde solution. After fixation, the tissues were subjected to sequential dehydration, clearing, and wax infiltration, followed by embedding in paraffin. A microtome was used to prepare tissue sections with a thickness of 5 μm.

Hematoxylin–Eosin (HE) staining was applied to the paraffin sections to visualize the liver's morphological structure and assess the extent of tissue damage. Masson staining was performed to stain collagen fibers blue; the stained area was then quantified to evaluate the degree of liver fibrosis.

For β‐galactosidase (β‐gal) staining, frozen liver sections (instead of paraffin sections) were used, as β‐gal serves as a specific marker for senescent cells. The proportion of β‐gal‐positive cells was observed and calculated to determine the level of liver aging.

#### 2.2.9 Western Blot Analysis

Liver tissue samples were harvested from mice, and an appropriate volume of protein lysis buffer was added to each tissue sample. The mixture was homogenized, and the resulting homogenate was centrifuged at 4°C for 15 min. The supernatant obtained from centrifugation was collected as the total protein extract from the liver tissue. Western blot analysis was carried out following standard procedures: gel electrophoresis, membrane transfer, blocking, primary antibody incubation, secondary antibody incubation, and chemiluminescent development. Images of the protein bands were captured using a gel imaging system, and the gray values of these bands were analyzed with Image J software. This analysis allowed for the quantitative assessment of expression levels of several target proteins: Fibrosis markers (α‐sma), aging markers (Sirt1, P53, P21, P16), NF‐κB signaling pathway components (IKKα/β, p‐IKKα/β, P65, p‐P65), Additionally, the ratios of (p‐IKKα/β)/(IKKα/β) and p‐P65/P65 were calculated.

#### 2.2.10 Cell Experiments

AML12 (mouse normal hepatocytes) and HepG2 (human hepatocellular carcinoma) cell lines were obtained from ATCC. AML12 cells were cultured in DMEM/F12 medium supplemented with 10% FBS, 1% ITS, 40 ng/mL dexamethasone, and 1% penicillin–streptomycin. HepG2 cells were maintained in MEM with 10% FBS and 1% penicillin–streptomycin at 37°C in 5% CO_2_. To induce senescence, cells were seeded in 6‐well plates (2 × 10^5^ cells/well) and treated with 20 μM Nutlin‐3a (MCE) for 48 h. Tgy‐N extract (prepared as in Section 2.2.1) was dissolved in sterile water and added at 1 mg/mL concurrently with Nutlin‐3a. Control cells received vehicle only. Protein expression of p16 and p21 was detected by Western blot following the procedure described in Section 2.2.8. SA‐β‐gal staining was performed and quantified according to the protocol in Section 2.2.7.

#### 2.2.11 Statistical Analysis

All experimental data were presented as mean ± SEM. Bioinformatics software was employed for the analysis of transcriptomic data, while SPSS 26.0 statistical software was used for general statistical analyses. One‐way analysis of variance (ANOVA) was utilized to compare data across multiple groups, and pairwise comparisons between groups were performed using Duncan's post hoc test. A *p*‐value less than 0.05 (*p* < 0.05) was considered to indicate a statistically significant difference.

## 3 Results

### 3.1 Major Chemical Components in Tgy‐N

After the purification process to obtain the extracts, the contents of major chemical components in Tgy‐N extracts were measured and are shown in Table 1.

**TABLE 1:** Major chemical constituents in the Tgy‐N.

Chemical constituents	Content (mg/g)
Tea polyphenols	286.9101 ± 1.4122
Catechins	137.8907 ± 4.7759
Tea polysaccharides	76.7312 ± 0.4746
Caffeine	48.4836 ± 6.9731
Free amino acids	21.6604 ± 2.5266
Thearubigins	9.2050 ± 1.3722
Theabrownins	7.6541 ± 0.7498
Total flavonoids	2.4429 ± 0.4640
Theaflavins	0.4992 ± 0.1044

*Note:* Data represent mean ± SEM of three replicates.

### 3.2 Composition of Tgy‐N by HPLC Analysis

The absolute contents of major catechins and gallic acid in Tgy‐N extract were determined by HPLC using authentic external standards. As shown in Table 2, the concentrations of EGCG, catechin (C), catechin gallate (CG), gallic acid (GA), and epigallocatechin (EGC) were 7.959, 5.786, 4.058, 1.665, and 4.913 mg/g, respectively. These quantitative data, together with the total polyphenol and total catechin contents presented in Table 1, confirm that Tgy‐N is rich in catechins.

**TABLE 2:** Absolute contents of major catechins and gallic acid in Tgy‐N extract determined by external standard HPLC.

Compound	Retention time (min)	Peak area (%)	Con (mg/g)
EGCG	17.456	7.66	7.959
C	6.428	1.28	5.786
CG	20.282	3.82	4.058
GA	4.343	1.11	1.665
EGC	6.254	0.35	4.913

*Note:* Data represent mean values of three replicates. EC, GCG, and ECG were not quantified due to the lack of commercial reference standards.

### 3.3 Tgy‐N Ameliorates Aging‐Associated Behavioral Impairments

An aging model was established in C57BL/6 mice via daily intraperitoneal injection of D‐gal. The mice were assigned to four groups: a control group received an intraperitoneal injection of 0.9% sodium chloride while being given water via intragastric administration; a model group received a daily intraperitoneal injection of 200 mg/kg D‐gal along with intragastric water; a low‐dose Tgy‐N (Tgy‐NL) group was administered 200 mg/kg/day D‐gal intraperitoneally in addition to 110 mg/kg/day Tgy‐N via gavage; and a high‐dose Tgy‐N (Tgy‐NH) group received the same D‐gal injection plus a higher dose of 550 mg/kg/day Tgy‐N intragastrically. Treatments were administered daily for 2 months (Figure 1A). Compared with the control group, D‐gal treatment led to a significant reduction in treadmill running time and distance; however, these behavioral deficits were reversed by Tgy‐NH (Figure 1B). Similarly, the decline in rotarod fatigue test performance induced by D‐gal was rescued by Tgy‐NH (Figure 1C). In contrast, while D‐gal treatment caused a significant decrease in grip strength relative to the control group, Tgy‐N had no notable impact on this parameter (Figure 1D). Results from metabolic cage experiments showed that D‐gal treatment significantly reduced the 24‐h energy expenditure (EE) of mice (Figure 1E). Notably, this reduction was primarily attributed to a marked decrease in EE during the dark phase (active period), whereas a slight EE increase was observed during the light phase (resting period). Tgy‐N treatment, especially at the high dose, reversed the D‐gal‐induced decrease in EE during the dark phase in a concentration‐dependent manner, while no significant improvement was observed during the light phase (Figure 1F).

**FIGURE 1:**
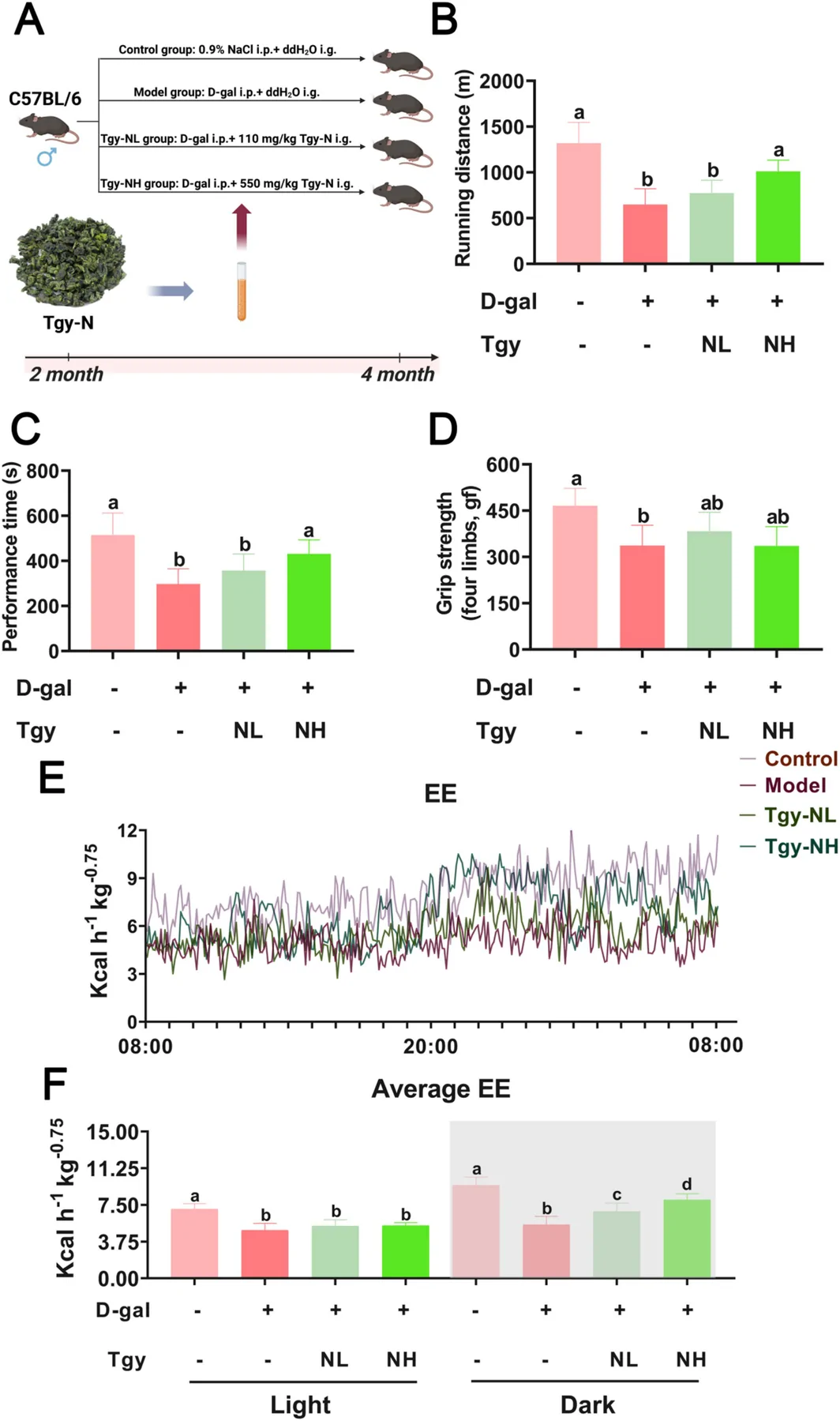
Schematic of modeling and Tgy‐N‐mediated improvement of behavioral traits. (A) Schematic illustration of the modeling procedure; (B) Treadmill test; (C) Rotarod fatigue test; (D) Grip strength test; (E) Metabolic cage test; (F) Quantitative analysis of metabolic cage test. For liver samples from the control, model, Tgy‐NL, and Tgy‐NH groups (*n* = 6), data are presented as mean ± standard error of the mean (SEM). Statistical analysis was conducted using one‐way analysis of variance (ANOVA) followed by post hoc Duncan's test. Different letters above the bars indicate statistically significant differences (*p* < 0.05) between groups.

### 3.4 Tgy‐N Exerts Hepatoprotection by Mitigating Oxidative Stress

Administration of Tgy‐N specifically improved D‐gal‐induced hepatic abnormalities without affecting other major organs, while also alleviating hepatic oxidative stress. Specifically, both Tgy‐NL and Tgy‐NH reduced the elevated liver‐to‐body weight ratio caused by D‐gal (Figure 2A), whereas no significant differences in the cardiosomatic index, renosomatic index, or pulmosomatic index were observed across all groups (Figure 2B–D). Blood samples collected from the orbital sinus and subsequent serum analysis revealed that Tgy‐NL and Tgy‐NH also decreased the increased serum levels of AST and ALT induced by D‐gal (Figure 2E,F). In the liver, D‐gal treatment resulted in reduced levels of SOD and GSH, along with increased MDA content; however, both Tgy‐NL and Tgy‐NH reversed these changes (Figure 2G–I).

**FIGURE 2:**
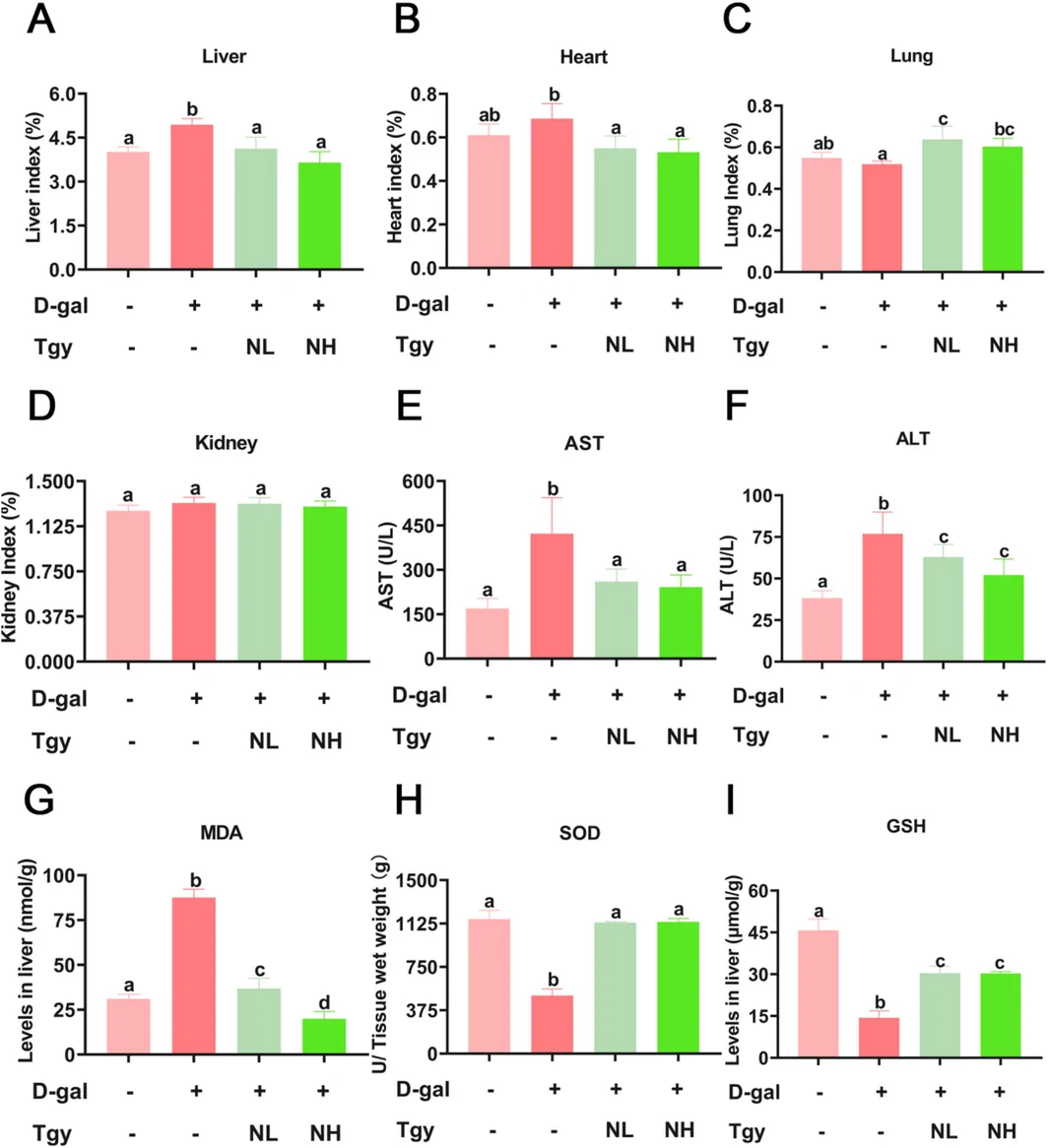
Tgy‐N reduces the hepatomatic index, mitigates oxidative stress, and protects liver function. (A) Hepatomatic index; (B) Cardiosomatic index; (C) Pulmosomatic index; (D) Renosomatic index; (E) Serum AST levels; (F) Serum ALT levels; (G) Hepatic SOD levels; (H) Hepatic MDA content; (I) Hepatic GSH levels. For liver samples from the control, model, Tgy‐NL, and Tgy‐NH groups (*n* = 6), data are presented as mean ± SEM. Statistical analysis was performed using one‐way ANOVA followed by post hoc Duncan's test. Different letters above the bars represent statistically significant differences (*p* < 0.05) between groups.

### 3.5 Tgy‐N Attenuates Hepatic Injury and Fibrosis

HE staining showed that D‐gal disrupted the hepatic lobule structure, caused disorganization of hepatocytes, and led to cytoplasmic loosening (damaged cells are indicated by white arrows); Tgy‐N treatment improved these pathological abnormalities (Figure 3A). Masson staining revealed extensive collagen fiber proliferation in the livers of D‐gal‐treated mice, which was reduced by Tgy‐N (Figure 3A); quantitative analysis confirmed that Tgy‐N exerted a dose‐dependent inhibitory effect on hepatic fibrosis (Figure 3B). Additionally, D‐gal treatment significantly increased the protein expression of α‐SMA, a marker of hepatic fibrosis; Tgy‐N reversed this upregulation in a dose‐dependent manner (Figure 3C,D). These results are consistent with the findings from Masson staining.

**FIGURE 3:**
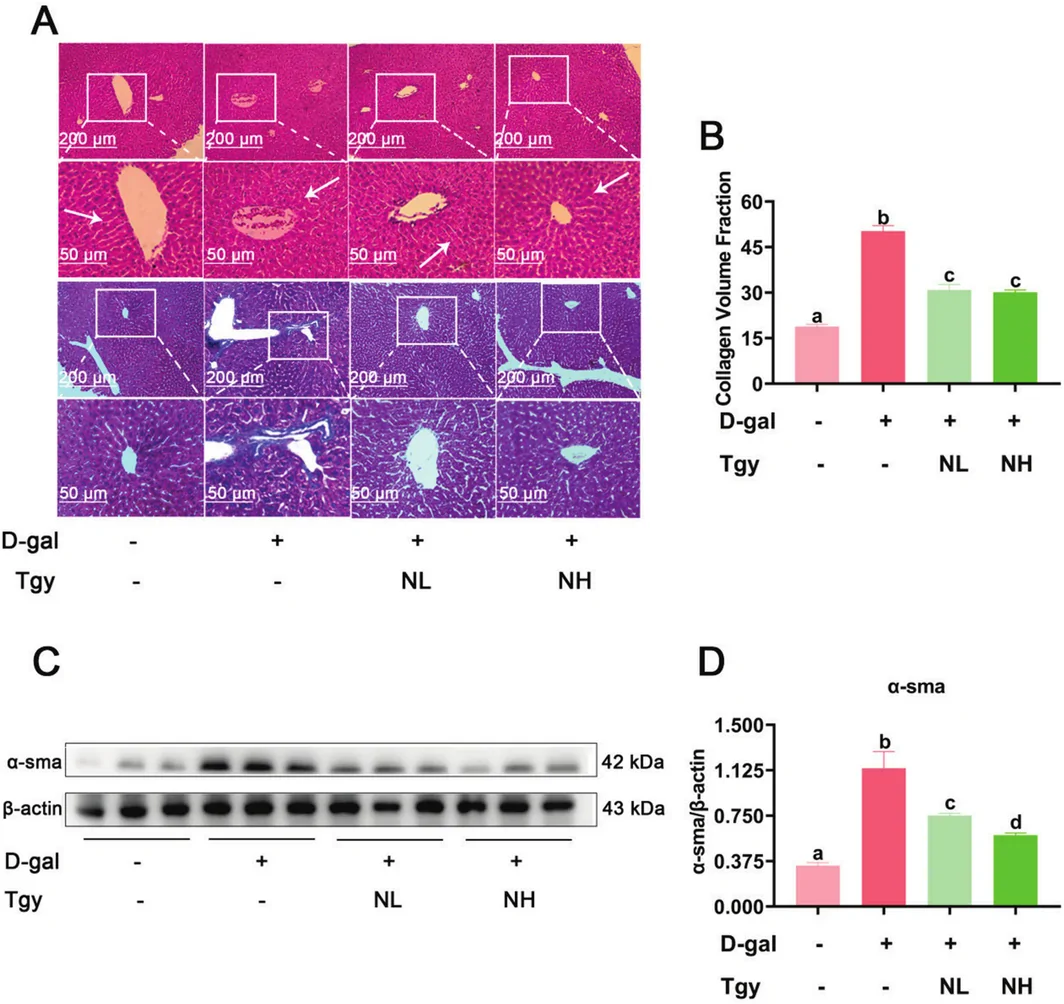
Tgy‐N alleviates hepatic injury and fibrosis. (A) Results of HE staining and Masson staining; (B) Quantitative analysis of Masson staining; (C) Protein expression of fibrosis markers; (D) Quantitative analysis of α‐SMA protein expression. For liver samples from the control, model, Tgy‐NL, and Tgy‐NH groups (*n* = 3), data are presented as mean ± SEM. Statistical analysis was conducted using one‐way ANOVA followed by post hoc Duncan's test. Different letters above the bars indicate statistically significant differences (*p* < 0.05) between groups.

### 3.6 Tgy‐N Retards D‐Gal‐Induced Hepatic Aging

Tgy‐N delayed D‐gal‐induced hepatic aging by regulating aging‐associated markers. Results showed that D‐gal increased hepatic β‐gal activity, which was inhibited by both Tgy‐NL and Tgy‐NH; the inhibitory effect of Tgy‐NH was more pronounced (Figure 4A,B). Furthermore, D‐gal treatment significantly upregulated the protein expression of aging markers (P16, P21, and P53) while downregulating the expression of the longevity‐related marker Sirt1. Tgy‐N reversed these changes in a dose‐dependent manner (Figure 4C–G), confirming its anti‐aging effect on the liver.

**FIGURE 4:**
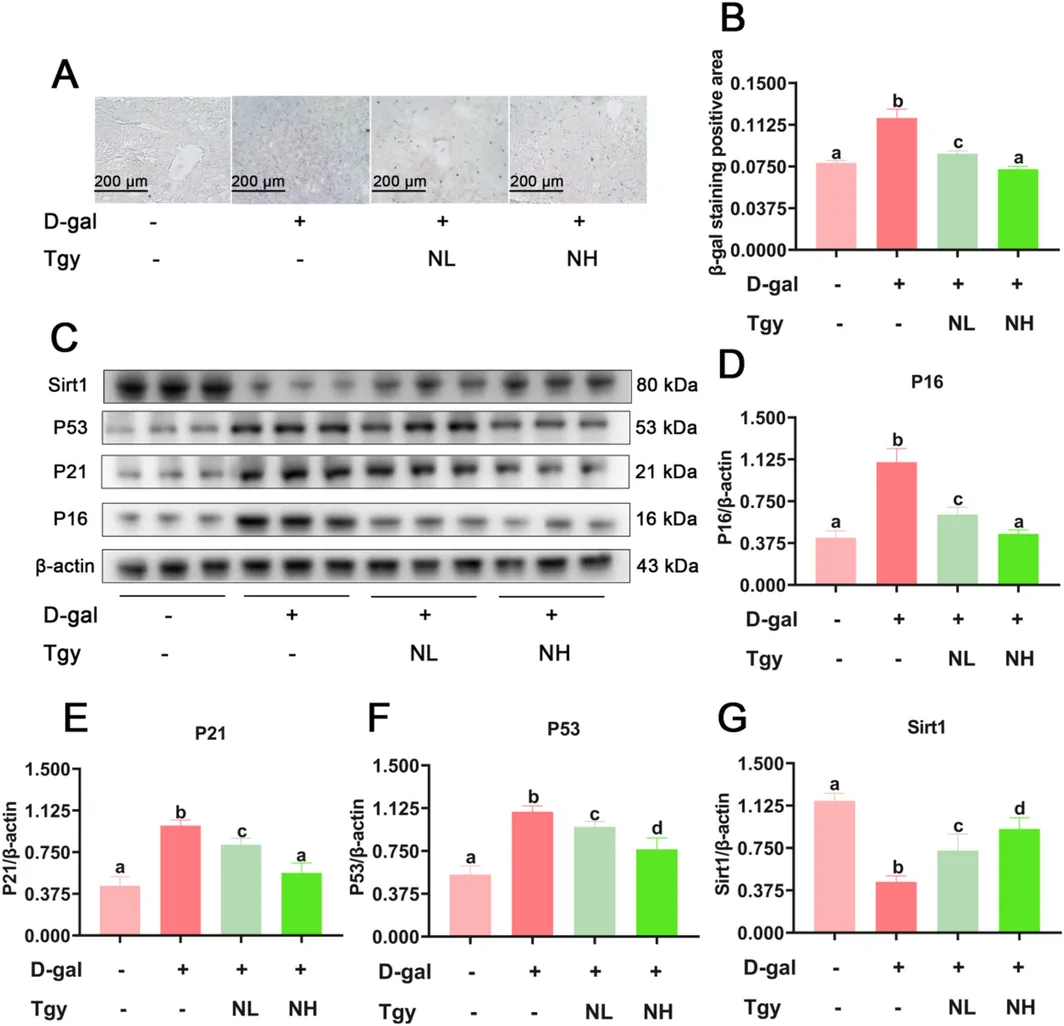
Tgy‐N retards D‐gal‐induced hepatic aging. (A) β‐gal staining; (B) β‐gal staining positive area; (C) Protein expression of aging markers; (D) Quantitative analysis of Sirt1 protein expression; (E) Quantitative analysis of P53 protein expression; (F) Quantitative analysis of P21 protein expression; (G) Quantitative analysis of P16 protein expression. For liver samples from the control, model, Tgy‐NL, and Tgy‐NH groups (*n* = 3), data are presented as mean ± SEM. Statistical analysis was performed using one‐way ANOVA followed by post hoc Duncan's test. Different letters above the bars represent statistically significant differences (*p* < 0.05) between groups.

### 3.7 Tgy‐N Suppresses Cellular Senescence Markers in AML12 and HepG2 Cells

To further validate the anti‐senescence effect of Tgy‐N at the cellular level, we established Nutlin‐3a‐induced senescence models in AML12 (mouse normal hepatocytes) and HepG2 (human hepatocellular carcinoma) cells. As shown in Figure 5A–J, Nutlin‐3a treatment (20 μM, 48 h) significantly upregulated the protein expression of p16 and p21 in both cell lines. Co‐treatment with Tgy‐N extract (1 mg/mL) markedly reduced the levels of p16 and p21 in a statistically significant manner. SA‐β‐gal staining further confirmed the anti‐senescence effect of Tgy‐N. Nutlin‐3a induced a marked increase in the percentage of SA‐β‐gal‐positive blue‐stained cells in both AML12 and HepG2 cultures, whereas Tgy‐N co‐treatment substantially decreased the number of senescent cells. These results provide direct cellular evidence that Tgy‐N protects against Nutlin‐3a‐induced senescence in both mouse and human hepatocyte‐derived cells, consistent with our in vivo findings in the aging mouse liver.

**FIGURE 5:**
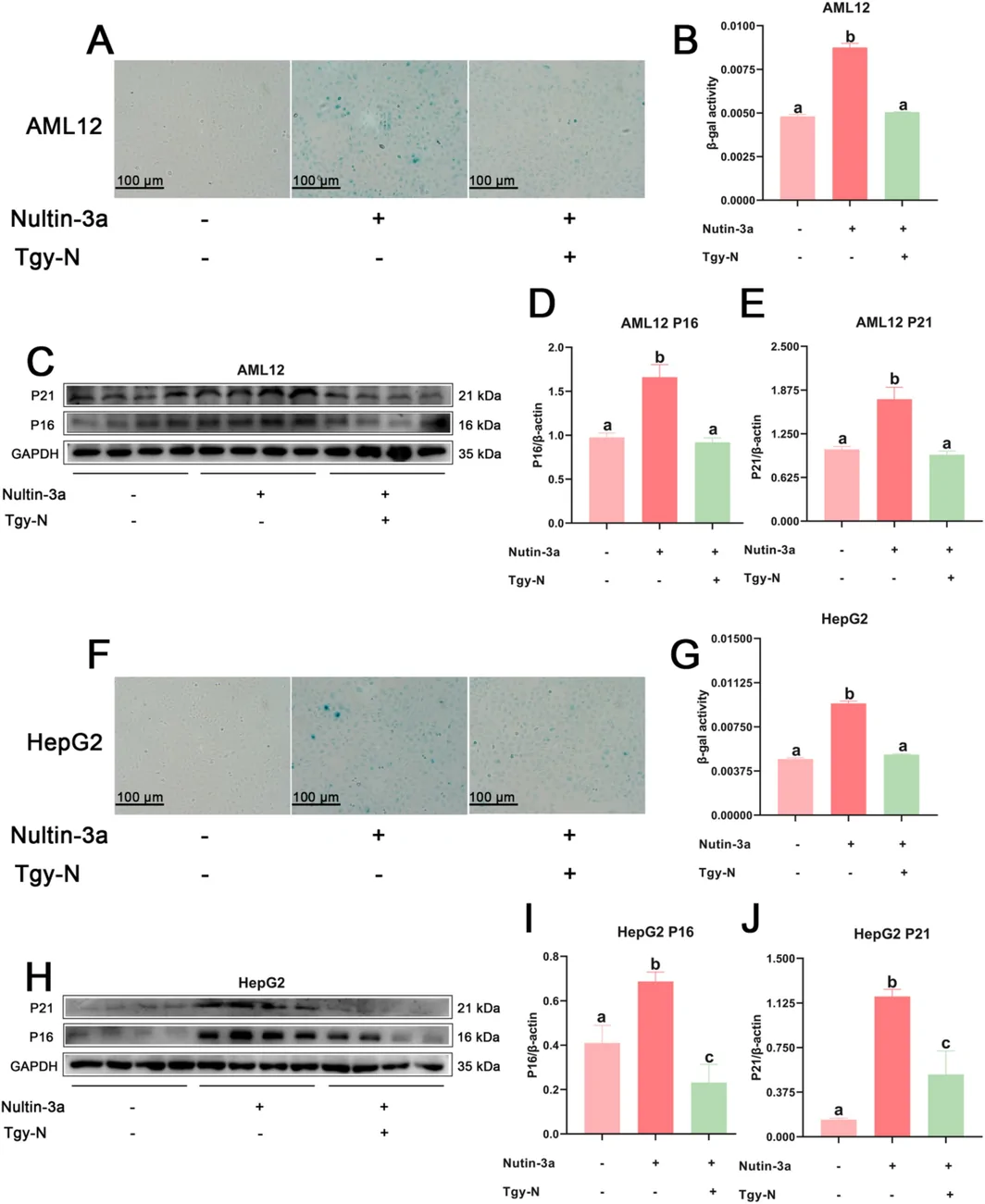
Tgy‐N reduces cellular senescence markers in Nutlin‐3a‐induced senescent AML12 and HepG2 cells. (A) SA‐β‐gal staining of AML12 cells. Scale bar = 100 μm. (B) Quantitative analysis of SA‐β‐gal‐positive AML12 cells. (C) Representative Western blots of p16, p21, and GAPDH in AML12 cells. (D) Quantitative analysis of p16/GAPDH ratio in AML12 cells. (E) Quantitative analysis of p21/GAPDH ratio in AML12 cells. (F) SA‐β‐gal staining of HepG2 cells. Scale bar = 100 μm. (G) Quantitative analysis of SA‐β‐gal‐positive HepG2 cells. (H) Representative Western blots of p16, p21, and GAPDH in HepG2 cells. (I) Quantitative analysis of p16/GAPDH ratio in HepG2 cells. (J) Quantitative analysis of p21/GAPDH ratio in HepG2 cells. Data are expressed as mean ± SEM. Statistical assessments were performed using one‐way ANOVA followed by post hoc Duncan's test. Different letters above the bars denote statistically significant differences between groups at the *p* < 0.05 level.

### 3.8 Hepatic Transcriptomics Identifies NF‐κB as a Key Mediator of Tgy‐N's Protective Effects

DEGs were screened using the criteria of *p* < 0.05 and |Log_2_FC| > 1. A total of 1604 DEGs were identified between the D‐gal and control groups (Figure 6A), and 3046 DEGs were detected between the D‐gal and Tgy‐NH groups (Figure 6B), with 216 DEGs overlapping between the two comparisons (Figure 6E). GO enrichment analysis (Figure S1) associated DEGs in both comparisons with developmental and cellular processes. KEGG enrichment analysis showed that the NF‐κB signaling pathway was significantly enriched in both the D‐gal vs. control and D‐gal vs. Tgy‐NH groups (Figure 6C,D). GSEA revealed that D‐gal treatment activated the NF‐κB pathway (Figure 6F), while Tgy‐NH treatment reversed this activation (Figure 6G).

**FIGURE 6:**
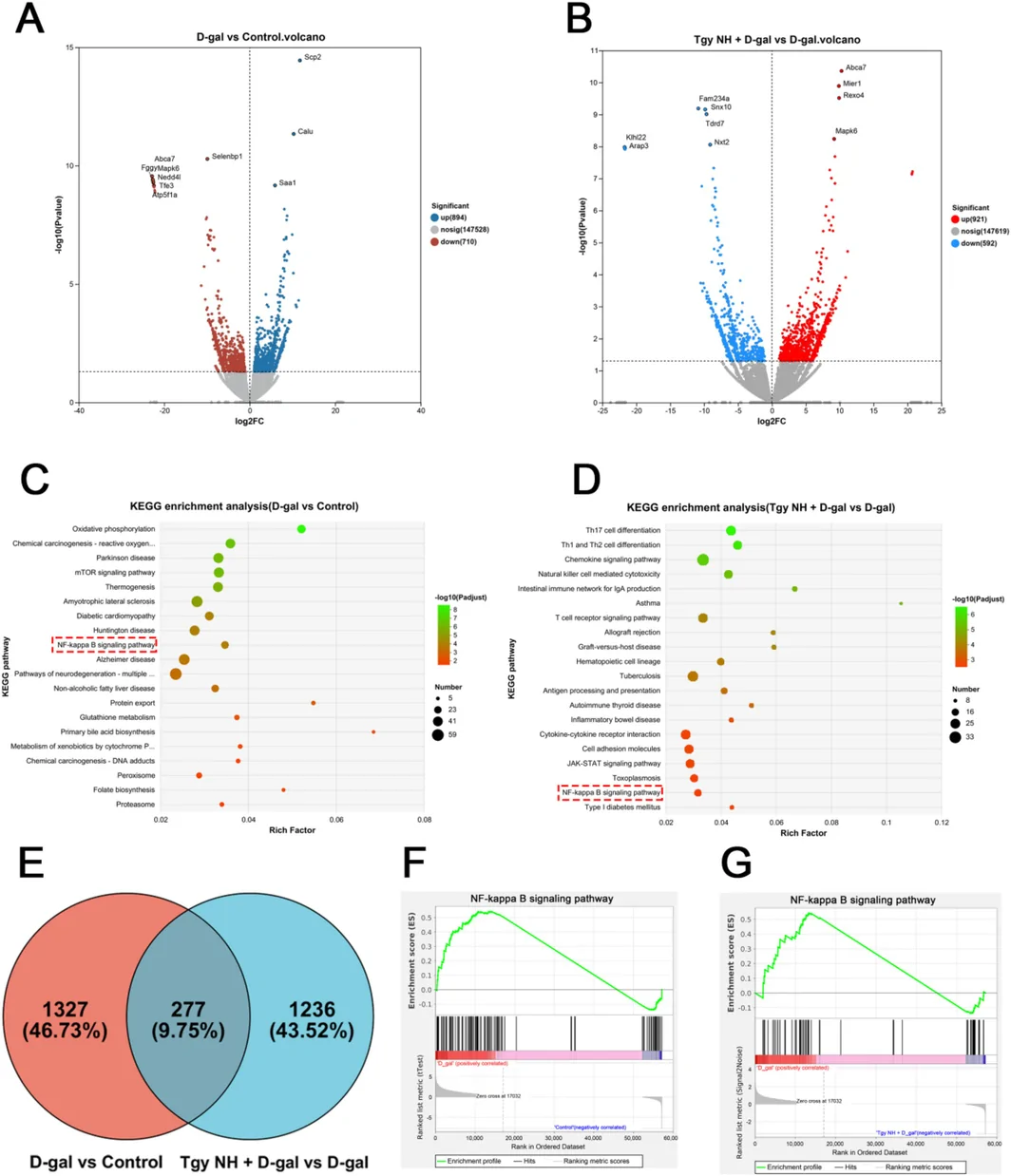
Hepatic transcriptomics indicates that Tgy‐N's hepatoprotective effect is associated with the NF‐κB signaling pathway. (A) Volcano plot of DEGs between the D‐gal group and the control group; (B) Volcano plot of DEGs between the D‐gal group and the Tgy‐NH group; (C) KEGG enrichment analysis results for the D‐gal group vs. control group; (D) KEGG enrichment analysis results for the D‐gal group vs. Tgy‐NH group; (E) Venn diagram; (F) GSEA results for the NF‐κB signaling pathway in the D‐gal group vs. control group; (G) GSEA results for the NF‐κB signaling pathway in the D‐gal group vs. Tgy‐NH group. Hepatic transcriptomics analysis was performed using samples from the control, D‐gal, and Tgy‐NH groups (*n* = 3). DEGs were screened with *p* < 0.05 and |Log_2_FC| > 1. Statistical analysis for enrichment analyses was based on hypergeometric tests, with significant enrichment defined by adjusted *p* < 0.05. GSEA results were evaluated using normalized enrichment scores (NES) and nominal *p* < 0.05.

### 3.9 Tgy‐N Suppressed the NF‐κB Pathway

Tgy‐N modulates the NF‐κB pathway to counteract the effects of D‐gal. KEGG enrichment analysis showed that the NF‐κB signaling pathway was enriched in both the D‐gal vs. control and D‐gal vs. Tgy‐NH groups (Figure 6C,D), while GSEA revealed that D‐gal activated this pathway and Tgy‐NH reversed the activation (Figure 6F–G). Experimental validation confirmed that D‐gal treatment activated the NF‐κB pathway. In contrast, Tgy‐N dose‐dependently suppressed the activation of this pathway (Figure 7A–D), which is consistent with the bioinformatics results.

**FIGURE 7:**
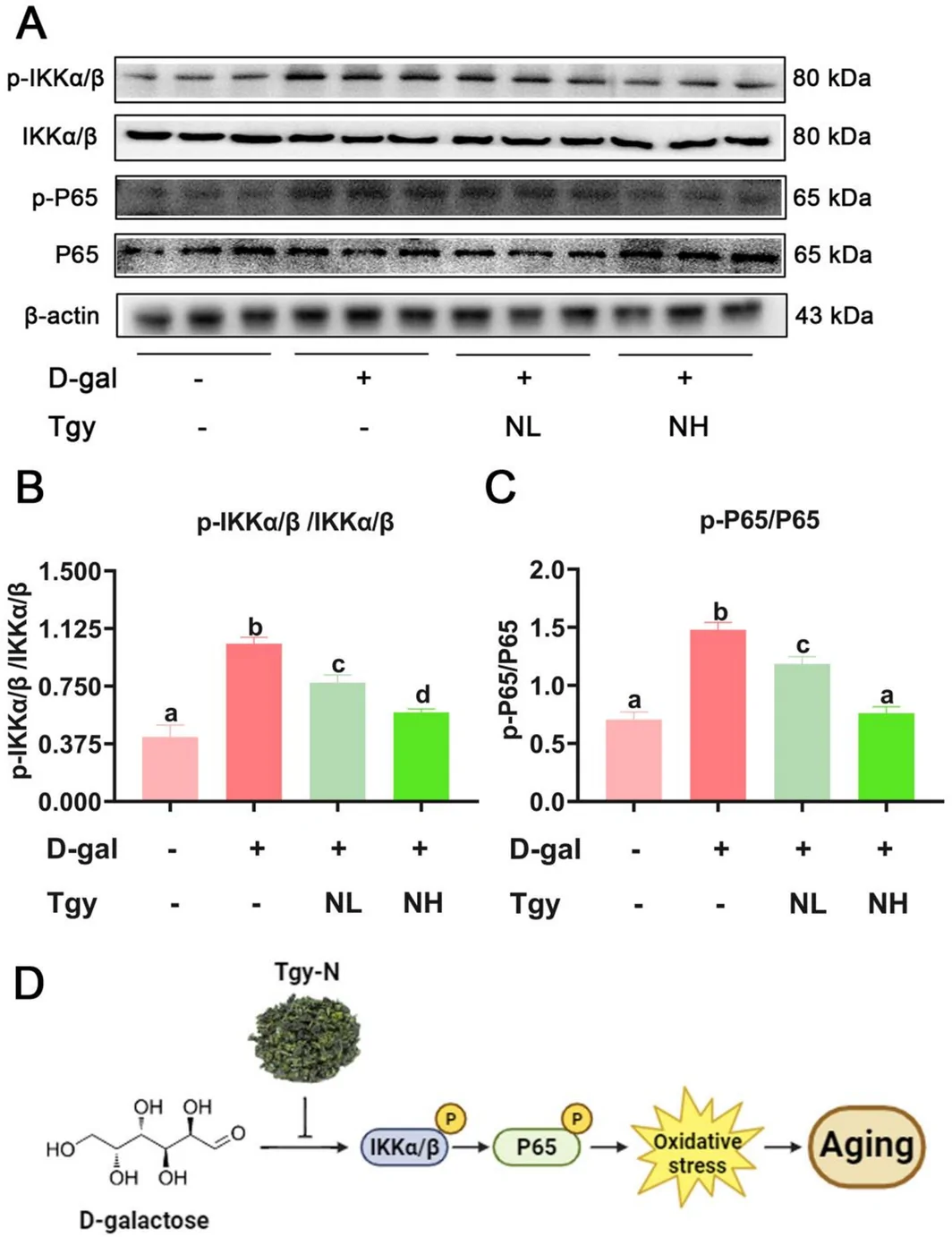
Tgy‐N suppresses the NF‐κB Pathway. (A) Protein expression of NF‐κB signaling components; (B) Quantitative analysis of the (p‐IKKα/β) (IKKα/β) ratio; (C) Quantitative analysis of the p‐P65/P65 ratio; (D) Schematic illustration of Tgy‐N's mechanism of action. For liver samples obtained from the control, model, Tgy‐NL, and Tgy‐NH groups (*n* = 3), data are expressed as mean ± standard error of the mean (SEM). Statistical assessments were conducted using one‐way analysis of variance (ANOVA), followed by post hoc Duncan's test for multiple comparisons. Distinct letters above the bars denote statistically significant differences between groups at the *p* < 0.05 level.

## 4 Discussion

Behavioral indicators serve as intuitive reflections of the D‐gal‐induced aging‐like phenotype in organisms (Kibitkina et al. 2025). Among these, the decline in exercise endurance and disruption of energy metabolism are typical characteristics of the aging process, which are closely associated with the degeneration of skeletal muscle function and the reduction in cellular energy metabolism efficiency (Garcia et al. 2025). In this research, D‐gal‐induced aging model mice exhibited a significant decline in exercise endurance (manifested by shortened exercise time and running distance). However, after intervention with Tgy‐N, the exercise endurance of mice improved in a concentration‐dependent manner.

Results from the metabolic cage experiment further revealed the regulatory role of Tgy‐N in aging‐related metabolic disorders. D‐gal treatment led to a significant reduction in 24‐h energy expenditure in mice, particularly during the dark phase (active period), while a slight increase in energy expenditure was observed during the light phase (resting period). This disruption in the circadian rhythm of energy metabolism may reflect aging‐associated mitochondrial dysfunction and reduced energy utilization efficiency, which can prompt compensatory metabolic responses (Gao et al. 2025; Steffan et al. 2025). Notably, Tgy‐N intervention, particularly at the high dose, reversed the D‐gal‐induced decrease in energy expenditure during the dark phase, restoring it to near‐normal levels without significantly affecting the light‐phase elevation. These findings suggest that Tgy‐N helps normalize the circadian pattern of energy metabolism in aging mice.

As the core organ responsible for metabolism and detoxification in the body, the liver's functional status is closely linked to the aging process (Salama et al. 2025). Enhanced hepatic oxidative stress and hepatocellular damage caused by aging not only directly induce liver dysfunction but also further exacerbate systemic aging through metabolic disorders (Singh et al. 2025). After D‐gal induction, the hepatomatic index of mice increased significantly. Tgy‐N intervention reversed this change in a dose‐dependent manner, while exerting no significant impact on the organ‐to‐body weight ratios of other organs such as the heart, lungs, and kidneys. This finding indicates that the protective effect of Tgy‐N is liver‐targeted. This targeting property may be related to the metabolic characteristics of the liver toward active components in tea: As the primary metabolic organ for drugs and exogenous substances, the liver can actively uptake polyphenolic compounds in tea (e.g., theaflavins, catechins) via transporters on the hepatocyte surface (such as organic anion‐transporting polypeptides, OATPs) (Ishihara et al. 2025). This uptake mechanism maintains a relatively high concentration of these compounds in the liver, thereby enabling targeted protective effects (Chen et al. 2025). Changes in serum liver function indicators (AST, ALT) further verified the liver protective effect of Tgy‐N.

Oxidative stress is a key driver of aging‐related liver injury. During the aging process, the decline in mitochondrial function in hepatocytes leads to increased production of ROS, while the activity of the antioxidant system (SOD, GSH) is attenuated. The imbalance triggers lipid peroxidation (with MDA as the main product), protein denaturation, and DNA damage, ultimately resulting in hepatocellular dysfunction (Li et al. 2025). In this research, D‐gal induction significantly increased hepatic MDA content and decreased SOD and GSH activities in mice. In contrast, Tgy‐N intervention markedly reversed these changes, indicating that Tgy‐N can alleviate aging‐related hepatic oxidative stress injury by enhancing antioxidant capacity.

Aging not only induces abnormalities in liver function and molecular levels but also causes significant histopathological alterations. Among these, hepatic lobule structural disorder, hepatocellular loosening, and fibrosis are typical features of aging‐related liver lesions. In the present research, HE staining, Masson staining, and protein level detection were combined to confirm the protective effect of Tgy‐N against hepatic pathological damage and fibrosis.

Results of β‐gal staining showed that D‐gal treatment significantly increased the number of β‐gal‐positive cells in mouse liver tissues, indicating the massive accumulation of senescent cells in the liver. However, Tgy‐N intervention reduced the number of β‐gal‐positive cells in a concentration‐dependent manner, suggesting that Tgy‐N can inhibit the accumulation of senescent cells in the liver. This result provides direct histological evidence for the anti‐aging effect of Tgy‐N. Western blot analysis further revealed the regulatory effect of Tgy‐N on senescence‐related proteins: D‐gal induction significantly upregulated the protein expression of P16, P21, and P53, while downregulating the expression of Sirt1 in the mouse liver.

P16 is a key senescence‐associated gene that binds to cyclin‐dependent kinase 4/6 (CDK4/6), inhibiting the formation of the Cyclin D‐CDK4/6 complex. This blocks retinoblastoma protein phosphorylation and E2F transcription factor dissociation, arresting cells in the G1 phase and inducing senescence (Lam et al. 2025). P21, transcriptionally regulated by P53, inhibits multiple Cyclin‐CDK complexes. It synergizes with P16 to reinforce G1 arrest, prevents DNA replication in the S phase, and regulates the senescence‐associated secretory phenotype (Da Silva et al. 2025). D‐gal activates P53 via the ataxia telangiectasia mutated/ATM and Rad3‐related pathway. Activated P53 upregulates P21 to exacerbate cell cycle arrest and induces apoptosis, while excessive activation promotes hepatocellular senescence (Jin et al. 2025). Sirt1 exerts NAD+ dependent deacetylase activity, deacetylating P53 and inhibiting NF‐κB, but its expression is suppressed by D‐gal. Tgy‐N intervention significantly reverses the D‐gal‐induced abnormal expression of these proteins (Liang et al. 2025). Nutlin‐3a is a specific MDM2 inhibitor that activates p53‐dependent senescence pathways and has been widely used to study cellular senescence. In our study, Tgy‐N treatment reduced p16/p21 expression and SA‐β‐gal positivity in Nutlin‐3a‐induced senescent AML12 and HepG2 cells. The consistent effects observed across mouse and human hepatocyte‐derived cells support the translational potential of Tgy‐N as an anti‐senescence agent (Zhu et al. 2025).

In this research, KEGG enrichment analysis via transcriptomics revealed that the NF‐κB signaling pathway was significantly enriched in the pairwise comparisons between groups. This conclusion was further reinforced by the results of GSEA. These findings confirm that the NF‐κB signaling pathway is a key target of Tgy‐N in intervening against aging‐related liver injury. The transcriptomic data integrate well with our biochemical and protein expression findings. KEGG enrichment and GSEA consistently identified the NF‐κB pathway as the most significantly altered cascade upon Tgy‐N treatment. This pathway‐level change corresponds with our observation that Tgy‐N reduces hepatic oxidative stress (elevated SOD/GSH, reduced MDA) and lowers the phosphorylation ratios of IKKα/β and p65. Collectively, the transcriptomic directionality aligns with the protein‐level suppression of NF‐κB signaling, supporting a model in which Tgy‐N alleviates D‐gal‐induced hepatic senescence by downregulating this pro‐inflammatory pathway. While catechin‐rich extracts from green or unroasted oolong teas are known to combat oxidative stress, our work uniquely demonstrates that Tgy‐N suppresses the canonical IKKα/β‐p65 NF‐κB axis in D‐gal‐induced hepatic senescence. This conclusion is supported by transcriptomic GSEA showing significant enrichment of the NF‐κB pathway, quantitative protein analysis revealing reduced p‐IKKα/β and p‐p65 ratios. This distinct mechanism has not been previously attributed to Nongxiang‐type Tieguanyin and extends our understanding of how roasting affects the bioactivity of oolong tea.

NF‐κB signaling pathway activation is a core feature of “inflammaging” in aging (Wei et al. 2025). Under senescent conditions, elevated intracellular oxidative stress and accumulated damaged organelles activate this pathway, driving transcription and secretion of inflammatory factors (Morgado et al. 2025). In its classical activation, external stress or inflammatory stimuli first bind membrane‐specific receptors. Via receptor‐mediated cascades, the cytoplasmic IKK complex (IKKα, IKKβ, IKKγ) is activated, triggering phosphorylation of IKKα (Ser176/180) and IKKβ (Ser177/181)—a key switch for pathway activation. Phosphorylated IKKβ then phosphorylates IκBα; the latter undergoes K48‐linked ubiquitination and 26S proteasome degradation, releasing the inhibited p65‐p50 dimer. This dimer translocates to the nucleus, binds κB sites in target genes, and initiates transcription of inflammatory/cell cycle genes (Zhang et al. 2021). Meanwhile, phosphorylated IKKα boosts p65's transcriptional activity by phosphorylating its Ser536 residue, enhancing co‐activator interaction (Wahl et al. 2024).

In this research, the livers of D‐gal‐induced senescent mice exhibited a significant increase in the expression of p‐IKKα/β, while the total levels of IKKα/β remained unchanged. This indicates that D‐gal triggers excessive activation of the NF‐κB pathway by specifically promoting the phosphorylation of IKKα/β. During aging, mitochondrial dysfunction in hepatocytes leads to massive accumulation of ROS (Almalki and Almujri 2024). ROS can induce the phosphorylation of both IKKα and IKKβ by oxidizing cysteine residues of the IKK complex and activating upstream transforming growth factor‐β‐activated kinase 1 (TAK1) (Xie et al. 2024). After Tgy‐N intervention, the abnormally high expression of p‐IKKα/β and phosphorylated p65 (p‐p65) in the livers of D‐gal‐induced senescent mice was significantly downregulated. The mechanistic link between oxidative stress reduction and NF‐κB inhibition is supported by multiple lines of evidence. Reactive oxygen species are known to activate the IKK complex via upstream kinases such as TAK1, leading to IκBα phosphorylation and subsequent p65 nuclear translocation. In our study, Tgy‐N treatment reduced hepatic MDA levels and restored SOD/GSH activities, indicating attenuated oxidative stress, which likely contributes to the observed suppression of p‐IKKα/β and p‐p65. Furthermore, Tgy‐N upregulated the longevity‐associated protein Sirt1, which can deacetylate p65 and reduce its transcriptional activity, providing an additional mechanism for NF‐κB inhibition. While additional cellular validation of upstream (e.g., TAK1 phosphorylation) and downstream events was not performed, the current multi‐level in vivo dataset provides sufficient mechanistic evidence supporting NF‐κB pathway inhibition as the key action of Tgy‐N. It is well‐established that the canonical NF‐κB pathway is activated through phosphorylation of IKKα/β at Ser176/180/177/181, followed by IκBα degradation and subsequent phosphorylation of p65 at Ser536, which represents the core functional activation axis. Accordingly, western blot analysis of p‐IKKα/β and p‐p65 ratios, combined with transcriptomic GSEA and KEGG enrichment, is widely accepted as sufficient to assess pathway modulation in aging and hepatoprotection studies. Previous research has demonstrated that p65 downregulation is a shared phenotype across multiple anti‐aging interventions in mouse liver, and similar markers (p‐IKKα/β, p‐p65, IKKα/β, p65) have been used to establish NF‐κB/IKK pathway inhibition in D‐gal‐induced liver aging (Wei et al. 2025; Elmansi et al. 2025). This regulatory effect exhibited a certain dose dependency, with the high‐dose Tgy‐N group showing a more pronounced improvement.

However, this research still has certain limitations. First, only a D‐gal‐induced aging model was used, and a natural aging model was not involved. We acknowledge a limitation of this study: the D‐galactose‐induced aging model primarily reflects oxidative stress‐related damage and does not fully recapitulate the complex and multifactorial nature of natural physiological aging. Therefore, our conclusions regarding the protective effects of Tgy‐N are specifically applicable to D‐gal‐induced senescence‐like phenotypes rather than natural aging per se. Future studies using naturally aged mouse models are warranted to confirm the translational relevance of our findings to human aging. The translational potential of these findings is noteworthy. As calculated in Section 2.2, the Tgy‐N doses used in this study (110 and 550 mg/kg/day in mice) are equivalent to approximately 5 and 25 g of dry Tgy‐N tea leaves per day for a 60 kg human. The lower dose of 5 g/day is within the range of typical daily tea consumption, suggesting that regular intake of Tgy‐N may offer liver‐protective benefits in aging populations. The higher dose of 25 g/day, while exceeding typical consumption, may be achievable through concentrated extracts or dietary supplements. Future clinical studies are needed to confirm efficacy and determine optimal dosing in humans. We also acknowledge that the Nutlin‐3a cellular experiments were designed to validate the anti‐senescence phenotype rather than the upstream oxidative stress or NF‐κB axis; therefore, the mechanistic link is supported by our in vivo data as associative evidence, and causal validation will be pursued in future studies.

## 5 Conclusions

This study demonstrates that Tgy‐N protects against D‐galactose‐induced hepatic senescence in mice. It improved exercise endurance, normalized energy metabolism, alleviated oxidative stress, ameliorated hepatic structural damage and fibrosis, and reversed senescence‐associated protein expression. Transcriptomic and Western blot analyses indicate that these effects are mediated through inhibition of the NF‐κB signaling pathway. The anti‐senescence activity was further validated in Nutlin‐3a‐induced senescent AML12 and HepG2 cells. These findings support the potential application of Tgy‐N as a natural hepatoprotective and anti‐aging agent.

## Author Contributions

**Weihong Chen:** conceptualization, investigation, funding acquisition, writing – original draft. **Mingmei Li:** methodology, validation, visualization, writing – review and editing. **Lingxiao Ye:** software, formal analysis, project administration. **Dongqin Huang:** data curation, supervision, resources. **Jianjia Xiao:** conceptualization, methodology, software, data curation. **Yuchao Sang:** investigation, validation, formal analysis, supervision. **Zizhen Wang:** funding acquisition, visualization, project administration, resources. **Jiehong Yang:** writing – original draft, writing – review and editing. **Chengyong He:** conceptualization, validation, visualization, writing – review and editing. **Jiabin Chen:** formal analysis, supervision, data curation, validation, visualization. **Zhenghong Zuo:** conceptualization, formal analysis, project administration, resources. **Xintan Chen:** conceptualization, investigation, validation, visualization, writing – review and editing, writing – original draft, project administration, resources.

## Funding

This research was supported by the Science and Technology Plan Project of Quanzhou City, Fujian Province, China (grant no. 2025QZNY104).

## Ethics Statement

The animal research protocol was approved by the Animal Ethics Committee of Xiamen University (approval no: XMULAC20170361).

## Consent

The authors have nothing to report.

## Conflicts of Interest

The authors declare no conflicts of interest.

## Supporting information


**Table S1:** HPLC method validation parameters.
**Figure S1:** GO enrichment analysis indicates that the action of Tgy‐N on the liver is associated with aging. (A) GO enrichment analysis of the D‐gal vs. Control group; (B) GO enrichment analysis of the D‐gal vs. Tgy‐NH group.

## Data Availability

The data presented in this research are available on request from the corresponding author Xintan Chen.
