# Supplementary material for: Protective Effects of Nongxiang‐Type Tieguanyin on the Liver of D‐Galactose‐Induced Aging C57BL/6 Mice via the NF‐κB Signaling Pathway
Source: Food Sci Nutr. 2026 Sep 29;14(10):e72396. doi: 10.1002/fsn3.72396 (PMC13621814; doi:10.1002/fsn3.72396)
Supplement: Supplementary file 1 [file FSN3-14-e72396-s001.docx]

**Supplementary data**

Figure 1 GO enrichment analysis indicates that the action of Tgy-N on the liver is associated with aging. A:GO enrichment analysis of the D-gal vs. Control group; B:GO enrichment analysis of the D-gal vs. Tgy-NH group.

Supplementary Table S1. HPLC method validation parameters

| Analyte | Linear range (μg/mL) | R^2^ | LOD (μg/mL) | LOQ (μg/mL) |
| --- | --- | --- | --- | --- |
| GA | 2-100 | ≥0.999 | 0.15 | 0.45 |
| EGCG | 2-100 | ≥0.999 | 0.18 | 0.55 |
| GC | 2-100 | ≥0.999 | 0.16 | 0.48 |
| C | 2-100 | ≥0.999 | 0.17 | 0.50 |
| EC | 2-100 | ≥0.999 | 0.12 | 0.38 |
| GCG | 2-100 | ≥0.999 | 0.20 | 0.60 |
| ECG | 2-100 | ≥0.999 | 0.19 | 0.57 |

The validation parameters listed in this table were cited from the GB/T 8313-2018 national standard method. These parameters were not re-determined in our laboratory because the quantitative HPLC analysis in this study was performed in accordance with this standard method.
